# Late-instar monarch caterpillars sabotage milkweed to acquire toxins, not to disarm plant defence

**DOI:** 10.1098/rspb.2023.2721

**Published:** 2024-02-21

**Authors:** Anja Betz, Robert Bischoff, Georg Petschenka

**Affiliations:** ^1^ Department of Applied Entomology, University of Hohenheim, 70599 Stuttgart, Germany; ^2^ KomBioTa—Center for Biodiversity and Integrative Taxonomy, University of Hohenheim and State Museum of Natural History, 70599 Stuttgart, Germany

**Keywords:** coevolution, *Danaus plexippus*, cardenolides, sabotaging, sequestration, latex

## Abstract

Sabotaging milkweed by monarch caterpillars (*Danaus plexippus*) is a famous textbook example of disarming plant defence. By severing leaf veins, monarchs are thought to prevent the flow of toxic latex to their feeding site. Here, we show that sabotaging by monarch caterpillars is not only an avoidance strategy. While young caterpillars appear to avoid latex, late-instar caterpillars actively ingest exuding latex, presumably to increase sequestration of cardenolides used for defence against predators. Comparisons with caterpillars of the related but non-sequestering common crow butterfly (*Euploea core*) revealed three lines of evidence supporting our hypothesis. First, monarch caterpillars sabotage inconsistently and therefore the behaviour is not obligatory to feed on milkweed, whereas sabotaging precedes each feeding event in *Euploea* caterpillars. Second, monarch caterpillars shift their behaviour from latex avoidance in younger to eager drinking in later stages, whereas *Euploea* caterpillars consistently avoid latex and spit it out during sabotaging. Third, monarchs reared on detached leaves without latex sequestered more cardenolides when caterpillars imbibed latex offered with a pipette. Thus, we conclude that monarch caterpillars have transformed the ancestral ‘sabotage to avoid’ strategy into a ‘sabotage to consume’ strategy, implying a novel behavioural adaptation to increase sequestration of cardenolides for defence.

## 1. Introduction

Upon mechanical injury, roughly 9% of all angiosperm plants exude latex, which often contains toxins and functions as a chemical and physical barrier against insect herbivores by poisoning them or gumming up their mouthparts [1–5]. Before taking up food, many plant-feeding insects therefore sabotage the latex-containing elongated cells (laticifers) running along the leaf veins and cut off leaf veins or petioles with their mandibles to drain the feeding site and to circumvent exposure to latex. This sabotaging behaviour has been observed in various, unrelated insects including caterpillars, beetles and katydids and likely represents a common response of insect species feeding on latex-bearing plants [6–10].

Sabotaging behaviour has been especially well studied in caterpillars of the monarch butterfly (*Danaus plexippus*, Lepidoptera: Nymphalidae: Danaini) feeding on toxic milkweed (*Asclepias* spp.) and represents a textbook example for the disarming of a plant defence trait by an insect [11–15]. Besides being sticky [16], the latex of many milkweed species contains high concentrations of cardenolides, sometimes far exceeding the cardenolide concentrations found in other plant tissues [17]. Cardenolides are potent toxins inhibiting Na^+^/K^+^-ATPase, an enzyme essential for numerous physiological processes in animals [18–20]. Importantly, monarchs cannot only cope with cardenolides by means of a resistant Na^+^/K^+^-ATPase [21] but are also famous for sequestering cardenolides in their body tissues as a defence against predators [22–24].

When sabotaging plants, caterpillars disable latex flow either by severing all minor laticifers supplying a small section on a leaf (trenching), or by cutting major veins, including petioles, that supply entire leave portions with latex (vein cutting) [25,26]. Notably, sabotaging behaviour in the milkweed butterflies (Danaini) is not restricted to *D. plexippus* but has also been observed in related species including *Danaus erippus, Danaus gilippus, Euploea core, Euploea crameri, Idea leuconoe, Lycorea cleobaea* and *Parantica sita* feeding on latex-containing plants of the Apocynaceae, Moraceae and Caricaceae [10,26–31]. Its widespread occurrence throughout the Danaini and reports from the less well studied but closely related Ithomiini [32,33] suggest that sabotaging may be an ancestral behavioural trait of the entire milkweed butterfly clade.

Interestingly, sabotaging behaviour in danaine caterpillars changes during larval development. While first- and second-instar larvae mainly trench, in older caterpillars the frequency of vein cutting increases. Isolating entire leaves from the latex flow by chewing a furrow in the petiole seems to be restricted to fifth- and sometimes fourth-instar milkweed butterfly caterpillars [10,26–28]. Traditionally, sabotaging behaviour in Danaini has been interpreted as an avoidance strategy to circumvent the toxicity and stickiness of milkweed latex [6,34–36]. However, *D. plexippus* adults and caterpillars clearly benefit from plant-derived cardenolides, which they can only acquire during the larval stage to use them as a defence against predators.

When watching late-instar *D. plexippus* caterpillars sabotaging *Asclepias curassavica* leaf petioles in the greenhouse, we never observed latex outflow and therefore speculated that caterpillars might drink exuding latex to acquire high amounts of cardenolides instead of avoiding it. Indeed, several authors have observed monarch caterpillars to occasionally ‘avidly imbibe’ and to ‘actively seek out and drink’ milkweed latex [12,37–39] but the function and consistency of latex drinking have never been addressed. Therefore, sabotaging behaviour in *D. plexippus* could also represent a co-option strategy in addition to the widely assumed disarming of a plant defence [8,35,37]. To evaluate the ecological function of sabotaging, we conducted experiments with *D. plexippus* caterpillars on *A. curassavica* and *Asclepias syriaca*, its two most important host plants globally [40]. While *A. syriaca* is the most important host plant in North America, *A. curassavica* is critical worldwide [40]. For comparison, we studied the caterpillars of the closely related common crow butterfly (*Euploea core*, Lepidoptera: Nymphalidae: Danaini), which also carry out sabotaging behaviour but do not sequester cardenolides, on the same host plants.

Specifically, we addressed the following questions: Is sabotaging a prerequisite for subsequent feeding? How do caterpillars react to latex on plants and when offered latex directly? Do monarch caterpillars remove substantial amounts of latex when cutting petioles? Do the caterpillars consume latex and absorb cardenolides during sabotaging? Does latex consumption in monarch caterpillars increase butterfly toxicity? and Does latex exposure decrease early-instar survival and is latex consumption limited to late instars?

## 2. Material and methods

### (a) Cultivation of plants

Seeds of *A. syriaca* were scarified with a scalpel blade to facilitate seed germination, whereas seeds of *A. curassavica* germinated well without scarification. Seeds of *A. incarnata*, which was only used to obtain caterpillars with low cardenolide levels as a specific requirement for sabotaging experiments on *A. syriaca* (see experiment iv), were also scarified. All seeds were embedded in moist tissue, placed in Petri dishes, sealed with breathable tape and stored at 28°C for 4 days in the dark. After germination, we planted seedlings in growing trays and transplanted them two weeks later into 11 × 11 × 12 cm (length × width × height) plastic pots (substrate 5, Klasmann-Deilmann GmbH mixed 3 : 1 with sand). Starting one week after transplanting, plants were fertilized weekly with a 0.2% dilution (v/v) of universal fertilizer (Wuxal Super N : P : K 8 : 8 : 6, Hauert MANNA Düngerwerke GmBH, Nürnberg, Germany). The bottom thirds of the *A. curassavica* and *A. incarnata* pots were constantly immersed in water, while pots with *A. syriaca* were left to drain after watering. All plants were grown in a greenhouse at 23–28°C/21–24°C (day/night). Ambient light was supplemented with artificial light. All plants were around three months old when used for experiments. Seeds were originally obtained commercially and subsequently propagated in our greenhouses/garden.

### (b) Butterfly rearing

*Danaus plexippus* (origin Portugal, stock provided by a local breeder) and *E. core* (origin Southeast Asia) were reared under greenhouse conditions (see above). Caterpillars of *D. plexippus* used for experiments were from a long-term colony maintained over multiple generations, while caterpillars of *E. cor*e were either offspring from freshly obtained adult specimens that were purchased commercially or were first- or second-generation offspring from commercially obtained specimens. We kept adult butterflies in 2 × 1 × 2 m flight cages and supplied them with a 10% sucrose solution and nectar plants (*A. curassavica*, *Lantana camara*, *Pentas lanceolata*). In addition, *E. core* caterpillars were provided with chopped seeds of *Heliotropium indicum and H. foertherianum* as a source of pheromonal precursors. Butterflies were sprayed with water eight times a day for 2 min by a fogging system (Micro Rain Systems, Altenburg, Germany) to increase humidity. For maintenance of butterfly colonies, caterpillars of both species were raised on potted *A. curassavica* plants under greenhouse conditions. For experiments, caterpillars were either reared on *A. curassavica, A. incarnata* or *A. syriaca* according to the experimental requirements (see below).

### (c) Experiments

#### (i) Is sabotaging a prerequisite for subsequent feeding?

**Figure 1.**
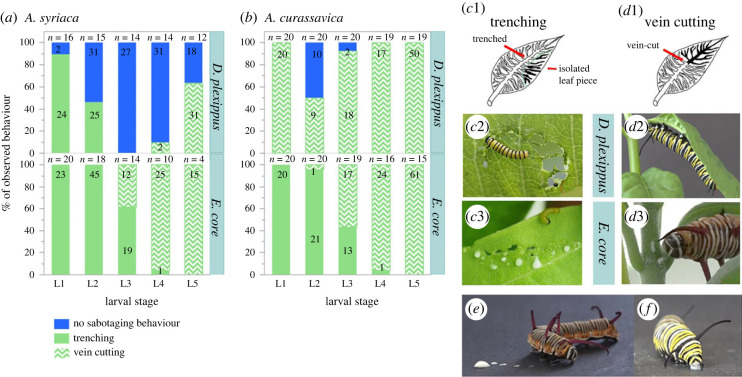
Sabotaging behaviour and latex drinking by caterpillars of the monarch butterfly (*D. plexippus*) and the common crow (*E. core*). Caterpillars (initial *n* = 20, per caterpillar species and plant species; total = 80) were raised either singly (monarchs) or in pairs of two (*Euploea*) on individual plants until pupation on *A. syriaca* or on (*b*) *A. curassavica*. Stacked bars represent the proportions of observed behaviours per instar, caterpillar species and hostplant species converted into percentages. Numbers in bars indicate the total observations recorded. Numbers above bars indicate the number of caterpillar individuals involved. Observed feeding behaviour was categorized into trenching (*c*1–3; i.e. cutting a series of minor leaf veins on the leaf blade, resulting in a trench), vein cutting (*d*1–3; i.e. cutting a single major leaf vein or the petiole) or no sabotaging behaviour (i.e. direct feeding on the leaf without signs of trenching or vein cutting; see electronic supplementary material, figure S1 for more detailed information on where sabotaging occurred on the leaves). (*e,f*) Caterpillar behaviour after artificial latex feeding: (*e*) *E. core* spitting out latex immediately; (*f*) *D. plexippus* drinking latex.

We placed one egg (*n* = 40) each on the upper third of approximately three-month-old *A. curassavica* (*n* = 20) and *A. syriaca* (*n* = 20) plants to observe the sabotaging behaviour of *D. plexippus* throughout larval development under ambient greenhouse conditions (see above). Sabotaging behaviour of caterpillars on both plant species, either directly observed or associated with a currently occupied caterpillar feeding site, was recorded once a day during daylight hours from caterpillar emergence to pupation. We differentiated sabotaging behaviour into trenching (cutting several leaf veins, resulting in a circular trench) and vein cutting (cutting only the main leaf vein, including the petiole; figure 1) according to Dussourd and Denno [25]. Observations of direct feeding without evidence of associated sabotaging behaviour were categorized as ‘no sabotaging’. We restricted our main analysis to either sabotaging or non-sabotaging (figure 1), but detailed information on sabotaging behaviour is provided in the supplementary material (electronic supplementary material, figure S1). We did not check whether caterpillars actually started feeding after observing ongoing sabotaging behaviour, but we had never observed sabotaging events without subsequent feeding.

A similar experiment with *E. core* was carried out in climate chambers (Fitotron HGC 1014, Weiss Technik, Reiskirchen, Germany) under increased humidity (27/24°C, 16/8 h light/ dark, 80/50% humidity) to prevent desiccation of the less robust early-instar caterpillars of *E. core*. Again, the initial sample size was *n* = 40 eggs, but due to space limitations in the climate chamber, two eggs were placed on each plant, resulting in *n* = 10 *A. syriaca* plants and *n* = 10 *A. curassavica* plants being sampled. Sabotaging behaviour was recorded once a day (during daylight hours) and classified as described for *D. plexippus* caterpillars. We note that although two caterpillars were placed on each plant, sabotaging behaviour could still be attributed to individual caterpillars. For statistical analysis, the response data collected were transformed to be binomial, i.e. all categories of sabotaging behaviour (trenching or vein cutting at different positions on the leaf) were assigned a value of ‘1’ and a value of ‘0’ was assigned if no sabotaging behaviour was observed. For the *E. core* experiment, statistical analysis was omitted because caterpillars sabotaged before each feeding event.

#### (ii) How do caterpillars react to latex on plants and when offered latex directly?

To compare the vein cutting behaviour of *D. plexippus* and *E. core* in detail, we observed vein cutting on the leaf petiole by last-instar caterpillars on *A. curassavica* (*E. core*: *n* = 11, *D. plexippus*: *n* = 12) and *A. syriaca* (*E. core*: *n* = 2, *D. plexippus*: *n* = 10). All observations were based on individual caterpillars and we also measured the duration of petiole cutting. For the latter, we assessed the time it took the caterpillars to sabotage the petiole until they turned around to start feeding from the tip of the leaf. For *D. plexippus*, we used caterpillars from our greenhouse colony and transferred them individually to unharmed plants in the laboratory. As *E. core* caterpillars are generally less active after transfer, observations for *E. core* were made in the greenhouse or climate chamber where the caterpillars were reared (conditions as described above). We note that we did not observe differences in petiole cutting duration and behaviour when observing additional caterpillars of *D. plexippus* under greenhouse conditions, therefore potential environmental differences did not appear to influence sabotaging behaviour. Five out of eleven caterpillars of *E. core* on *A. curassavica* sabotaged at the midrib of the leaf blade, whereas *D. plexippus* exclusively cut furrows in the petiole. We also filmed a subset of caterpillars (Olympus OM-D E-M1 Mark III) to illustrate typical caterpillar behaviour.

In addition to observations on real plants, we hand-fed a set of *A. curassavica* reared fifth (=last)-instar caterpillars (*n* = 12) of both caterpillar species from our colonies with 6 µl of *A. curassavica* latex in the laboratory to facilitate observations on caterpillar behaviour to direct latex exposure, and again videotaped their response. Hand feeding was performed by holding the caterpillar with one hand and pipetting latex onto the mandibles with the other hand. A volume of 6 µl of latex was found to be representative when petiole cutting was mimicked with a razor blade on additional, intact *A. curassavica* plants (twice per plant on an upper and a lower leaf; i.e. two averaged measurements per plant, *n* = 10 plants) and the outflowing latex was measured with glass capillaries (6.4 ± 0.5 µl, mean, s.e.).

#### (iii) Do monarch caterpillars remove substantial amounts of latex when cutting petioles?

To evaluate the amount of latex removed by monarch caterpillars during petiole cutting, individual *A. curassavica*-reared fifth-instar monarch caterpillars (*n* = 9) were placed on three-month-old *A. curassavica* plants. After petiole cutting, caterpillars were removed and distal leaf portions were cut off 5 cm from the tip with a razor blade. Outflowing latex was collected on a pre-weighed filter paper from both leaf portions (remaining leaf base and tip) and weighed immediately on a microbalance. For comparison, we used leaves of the same size and age from the same batch of intact *A. curassavica* plants (*n* = 10) and collected latex as described above.

#### (iv) Do the caterpillars consume latex and absorb cardenolides during sabotaging?

We tested whether fifth-instar caterpillars of *D. plexippus* actively drink latex during petiole cutting by chemical analysis of cardenolides in caterpillar regurgitates (electronic supplementary material, figure S2). Caterpillars of *D. plexippus* were raised from eggs on *A. syriaca* (*n* = 10) and *A. incarnata* (*n* = 10) plants under greenhouse conditions. After reaching the fifth instar, we induced regurgitation in actively feeding caterpillars by tweaking the caterpillars' abdomen with tweezers. Regurgitates were collected (either all or a fraction) using glass capillaries and sample volumes were recorded. These samples were used as blanks. Next, we transferred *A. syriaca*-reared caterpillars to uninjured *A. curassavica* plants and *A. incarnata*-reared caterpillars to uninjured *A. syriaca* plants (one caterpillar per plant). We used *A. syriaca* and *A. incarnata* for rearing caterpillars since the cardenolides produced by these milkweed species differ structurally from the cardenolides found in *A. curassavica* or *A. syriaca*, respectively. Consequently, cardenolides ingested during vein cutting can be easily differentiated chromatographically from cardenolides present in the previous host plant. All caterpillars transferred to the experimental plants started petiole cutting within 1 h. When petiole cutting was completed and the caterpillars started to move towards the leaf tip, a second regurgitate sample was collected before the caterpillars started to feed to prevent uptake of cardenolides from the leaf material. All samples were extracted and the cardenolide content was quantified using high performance liquid chromatography (HPLC; see below for methods of extraction and quantification). In parallel, we carried out a similar experiment with *E. core* to test whether caterpillars avoid oral uptake of latex during vein cutting. We used caterpillars raised from eggs on *A. curassavica* (*n* = 8) since *E. core* do not grow well on *A. syriaca*. Before the experiment, fifth-instar caterpillars were transferred to uninjured *A. curassavica* and samples were collected as described above. Due to the tendency of *E. core* to chew furrows above the petiole (observed in six out of eight caterpillars), we also included caterpillars that carried out vein cutting within the proximal third of the leaf. Across all experiments, regurgitate sample volumes ranged from 5 to 30 µl (16.01 ± 0.75 µl, mean ± s.e.).

#### (v) Does latex consumption in monarch caterpillars increase butterfly toxicity?

To evaluate the effect of latex drinking in caterpillars on overall toxicity of adult monarch butterflies (i.e. sequestered cardenolides), we raised caterpillars either (1) on detached *A. curassavica* leaves (see below for details), or (2) on detached leaves with additional supply of *A. curassavica* latex by hand feeding the caterpillars (initially *n* = 20 per diet). Both groups of caterpillars were reared on freshly detached *A. curassavica* leaves after excision of the midrib to maximize latex removal and mimic feeding on sabotaged leaves. As no latex exudation was visible on the cut edges (electronic supplementary material, figure S3), we assume that latex removal was complete. A maximum of two leaves per plant per day were collected from the donor plants and leaves were removed with a razor blade to avoid cardenolide induction by harvesting [41]. Caterpillars were individually maintained in plastic containers (125 ml until L1–L4, 500 ml in L5) lined with moist filter paper and raised from eggs to butterflies in the greenhouse. For (2), caterpillars were hand-fed with 6 µl of *A. curassavica* latex three times per day using a pipette. Supplementation with latex was limited to the fifth instar since caterpillars showed a marked increase in the frequency of petiole cutting during the last instar (see electronic supplementary material, figure S1). As mentioned above, 6 µl was found to be a typical volume of latex-outflow and three petiole cutting events per day represent a conservative estimate of the daily petiole cutting frequency in fifth-instar monarch caterpillars. Resulting pupae were transferred to the laboratory for hatching. After wing hardening, butterflies were frozen at −80°C. Butterflies were stored at −80°C, freeze-dried (Alpha 2–4 LDplus, Martin Christ, Osterode, Germany), weighed on a microbalance (0.001 g; Cubis II, Sartorius Corporate Administration GmbH, Göttingen, Germany), extracted, and analysed via HPLC-diode array detection (DAD), as described below. Limited sample sizes (seven butterflies on detached leaves, six butterflies on detached leaves with latex supply) in this experiment are due to high mortality of caterpillars reared in containers, which was probably due to adverse environmental conditions (high temperatures) in the greenhouse.

#### (vi) Does latex exposure decrease early-instar survival and is latex consumption limited to late instars?

In a separate experiment using an independent set of caterpillars, we assessed the effect of plant latex on caterpillar survival by rearing caterpillars on detached leaves, with and without additional latex supply. Survival data collected in this experiment were compared with caterpillar survival data on intact plants collected in experiment i. This comparison was made to re-evaluate previous findings suggesting that latex causes high mortality rates in first-instar caterpillars [42]. In addition, we investigated the response of caterpillars of all instars to latex pipetted to the mouthparts to test for behavioural changes during larval development. Caterpillars of *D. plexippus* were raised from eggs in plastic containers lined with moist filter paper (cups of 125 ml until L3, 500 ml for L4–L5) on detached leaves of *A. syriaca* or *A. curassavica* (27/24°C, 16/8 h light/ dark, 60/0% humidity in a Fitotron^®^ HGC 1014 chamber, Weiss Technik, Reiskirchen, Germany). On both plant species, half of the caterpillars were fed with latex of the corresponding plant once a day (L1–L2 = 1 µl, L3 = 2 µl, L4 = 4 µl, L5 = 6 µl). The other half was fed an according volume of water (initially, *n* = 20 for each treatment). Latex or water feeding was carried out with a pipette. The behaviour upon latex or water exposition and survival of caterpillars were recorded across caterpillar development.

#### (d) Chemical analysis

*Extraction of caterpillar regurgitates for chemical analysis.* We collected samples (in glass capillaries) in 2 ml screw-cap micro tubes containing 0.9 g zirconia beads (2.3 mm, Carl Roth GmbH, Karlsruhe, Germany) and 1 ml methanol. Samples were homogenized with a FastPrep homogenizer (MP Biomedicals, Eschwege, Germany) for two cycles of 45 sec at 6.5 m s^−1^. After 3 min of centrifugation (16 000 g at 22°C; 5417R, Eppendorf, Hamburg, Germany), we transferred the supernatant into new 2 ml screw-cap micro tubes and repeated the procedure once. Subsequently, pooled samples were evaporated to dryness in a vacuum centrifuge (RVC 2–25 CDplus, Martin Christ, Osterode, Germany). Finally, dried residues were dissolved in 100 µl methanol, agitated in the FastPrep instrument (45 sec; 6.5 m s^−1^) and centrifuged (3 min, 16 000 g, 22°C). Subsequently, supernatants were filtered into HPLC vials using Rotilabo syringe filters (nylon, 0.45 µm pore size, Ø 13 mm, Carl Roth GmbH & Co.KG, Karlsruhe, Germany).

*Extraction of butterflies for chemical analysis.* Freeze-dried samples were transferred into 15 ml centrifuge tubes containing two ceramic beads (6.35 mm, MP Biomedicals, Graffenstaden, France) and 2 ml of methanol. The samples were homogenized in the FastPrep instrument as described above. After centrifugation for 10 min (1000 g at 22°C; Labofuge 400R, Heraeus Instruments, Osterode, Germany), we transferred 2 ml supernatants into a glass test tube. Extraction of samples was repeated once with 2 ml methanol. Pooled supernatants (4 ml) were evaporated to dryness in a vacuum centrifuge (RVC 2–25 CDplus, Martin Christ, Osterode, Germany). Dried residues were dissolved in 500 µl methanol and transferred into a 2 ml screw cap micro tube. This procedure was repeated twice using an ultrasonic bath during the last round to completely dissolve any residue. The 1.5 ml of pooled sample was once more evaporated to dryness in the vacuum centrifuge. Finally, samples were dissolved in 500 µl methanol, homogenized (45 s; 6.5 m s^−1^), centrifuged (3 min, 16 000 g, 22°C) and filtered (nylon filter 0.45 µm, Ø 13 mm) into HPLC vials.

*Cardenolide quantification via HPLC.* We quantified the cardenolide content of the samples using an Agilent Infinity 1260 II HPLC system (Agilent Technologies, USA) equipped with an EC 150/4.6 NUCLEODUR C18 Gravity column (3 µm particle size, 150 mm × 4.6 mm, Macherey-Nagel, Düren, Germany). We injected 15 µl of sample, which was eluted at a constant flow rate of 0.7 ml min^−1^ using a water/acetonitrile gradient as follows: 0–2 min 16% acetonitrile, 25 min 70% acetonitrile, 30 min 95% acetonitrile, 35 min 95% acetonitrile, 37 min 16% acetonitrile, 10 min reconditioning at 16% acetonitrile. Peaks were detected at 218 ± 4 nm with a DAD. Absorbance spectra were recorded between 200 and 400 nm. Peaks with a characteristic symmetric absorption spectrum with a maximum between 218 and 222 nm were classified as cardenolides [43]. Cardenolides were quantified at 218 ± 4 nm using a digitoxin (a standard cardenolide) calibration curve (5, 10, 25, 50, 100, 250, 500, 750 and 1000 µg ml^−1^). All cardenolide peaks in one sample were summed up to calculate total cardenolide concentrations.

#### (e) Data analysis

Statistical analysis was performed using SAS v.9.4 software (SAS Institute, Cary, NC, USA). For statistical codes and output, see the statistical documentation in the electronic supplementary material. *P*-values < 0.05 were considered statistically significant. Data collected in this study were either metric (experiments iii–v) or binomial (experiments i, ii, vi). Metric data were tested for within-group normality using the Shapiro–Wilk test. Homogeneity of variances was tested using the folded *F*-test. Where variances were equal, groups were compared using a *t*-test (experiment iii, and cardenolide concentrations in experiment v); where variances were unequal, Satterthwaite's *t*-test was used (total cardenolides in experiment v). Where within-group normality was rejected, the Wilcoxon two-sample rank test was used (experiment iv).

Binomial data were analysed using generalized linear mixed (GLMM) models assuming a logit link function (experiments i, vi); residual and QQ-plots were checked for homogeneity of variances and normality. Overdispersion of the GLMMs was rejected if generalized chisq./DF was close to 1. In these experiments, caterpillars were tested multiple times during their larval development, so a repeated random effect was fitted with the subject level ‘caterpillar individual’. The assumed correlation structure (covariance structure) was autoregressive. In experiment i, no interaction of larval stage x host plant was included in the model because the model failed to converge, probably due to overparameterization. However, the linear predictors of the model reflected the raw data well. For the data from experiment vi (survival), only neonate caterpillars were included in the analysis, as they are thought to be particularly vulnerable to latex. In addition, no covariance structure was assumed as the event of interest (death) can only occur once. For experiment ii, Fisher's exact test was used. No outliers were excluded for all analyses. Graphs of our data were generated using JMP Pro v.16.1 (SAS Institute, Cary, NC, USA).

## 3. Results

### (a) Is sabotaging a prerequisite for subsequent feeding?

To test whether sabotaging is essential for subsequent feeding and therefore an adaptation to cope with latex, we examined how consistently sabotaging behaviour was performed across all developmental instars of both caterpillar species. Our results show that sabotaging preceded each feeding event of *Euploea* on both milkweed species (*A. curassavica* and *A. syriaca*), whereas monarch caterpillars sabotaged milkweed inconsistently (figure 1*a,b*). Specifically, the frequency of sabotaging by monarch caterpillars varied between larval stages (*F*_4,212.9_ = 12.44; *p <* 0.001) and host plant species (*F*_1,76.02_ = 27.3; *p <* 0.001). Especially on *A. syriaca*, sabotaging behaviour of monarch caterpillars became less frequent after the first instar. During the first instar, monarch caterpillars sabotaged in 90% of the observations, whereas sabotaging behaviour was found in only 46% of the second-instar observations and was completely absent in the third-instar caterpillars (figure 1*a*). On *A. curassavica*, monarch caterpillar sabotaging was more consistent (92–100% of observations in the first, third, fourth and fifth instars) but also absent in 50% of the observations of second-instar caterpillars (figure 1*b*; for exact locations of sabotaging see electronic supplementary material, figure S1).

### (b) How do caterpillars react to latex on plants and when offered latex directly?

Using additional last-instar caterpillars, we carried out detailed observations on petiole cutting behaviour on intact plants of both milkweed species. Interestingly, we found strikingly different behaviours between monarchs and *Euploea*. Caterpillars of *Euploea* always spat out latex multiple times close to the furrow and dabbed their mandibles on the plant stem to remove latex (*n* = 11 on *A. curassavica*; *n* = 2 on *A. syriaca*). During one petiole cutting event on *A. curassavica*, *Euploea* interrupted for latex removal nine times on average (±3.39 s.e., *n* = 7). By contrast, we never observed latex-spitting in monarchs (*n* = 12 on *A. curassavica*; *n* = 10 on *A. syriaca*; see electronic supplementary material, videos S1 and S2).

These differences in latex handling were also observed when we applied *A. curassavica* latex with a pipette to the mouthparts of last-instar caterpillars of both species in the laboratory (i.e. a single application of 6 µl per caterpillar, *n* = 12 for each species). In agreement with our previous observations on whole plants, 10 out of 12 caterpillars of *Euploea* spat out latex immediately (figure 1*e*). By contrast, all monarch caterpillars (*n* = 12) imbibed latex instantly (Fisher's exact test, *p* < 0.001, response variable ‘latex drinking: yes/no’, figure 1*f*; see electronic supplementary material, video S3). In accordance with the observed behavioural differences, *Euploea* caterpillars took over six times longer to cut petioles on *A. curassavica* plants than monarchs (Wilcoxon, *T* = 198, *z =* 4.032, *p* < 0.001). Comparisons on *A. syriaca* revealed the same trend but statistical analysis was not possible due to limited sample size (see the electronic supplementary material for details).

### (c) Do monarch caterpillars remove substantial amounts of latex when cutting petioles?

To test how efficiently caterpillars of *D. plexippus* remove latex during petiole cutting, we compared latex outflow of intact (*n* = 10) and sabotaged *A. curassavica* leaves (*n* = 9) and found that petiole cutting reduced latex outflow substantially (0.72 ± 0.13 mg fresh weight versus 0.1 ± 0.02 mg fresh weight, mean ± s.e.; *t*_17_ = −13.22, *p* < 0.001).

### (d) Do the caterpillars consume latex and absorb cardenolides during sabotaging?

**Figure 2.**
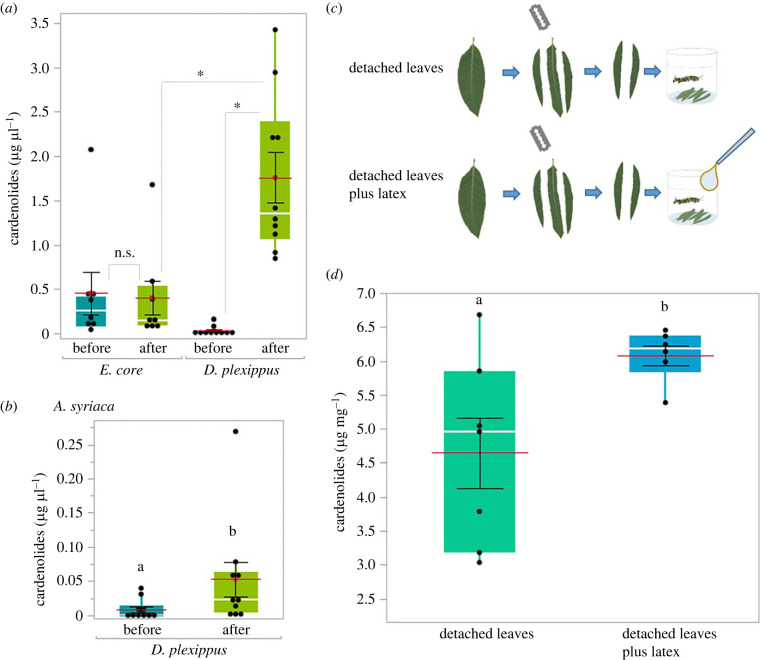
Cardenolide concentrations in caterpillar regurgitates before and after sabotaging and insect tissues. (*a*) Cardenolide concentration in regurgitates of *D. plexippus* and *E. core* fifth-instar caterpillars before and after vein cutting on *A. curassavica*. (*b*) Cardenolide concentration in foregut content of *D. plexippus* before and after vein cutting on *A. syriaca*. (*c*) Experimental setup: caterpillars were raised from egg until pupation on latex-free (midrib removed) detached leaves of *A. curassavica* (top) or on the same diet but hand-fed with latex during the last (fifth) instar (3 × 6 µl per day). (*d*) Concentration of sequestered cardenolides in adult butterflies between experimental diets. Different letters and asterisks above bars indicate significant differences between treatments. Boxes indicate interquartile ranges and white lines show medians. Black dots designate individual data points. Red lines represent means and black lines indicate SE. Note that the data shown in (*a*) and (*b*) were analysed using the Wilcoxon two-sample rank test, but means and SE are still shown to describe the data.

Next, we tested the ingestion of latex and cardenolides by chemical analysis of caterpillar regurgitates (foregut contents; for experimental setup see electronic supplementary material, figure S2) for both caterpillar species. Regurgitates of fifth-instar monarch caterpillars (*n* = 10) harvested right after petiole cutting on *A. curassavica* had 10-fold higher cardenolide concentrations compared to caterpillars of *Euploea* (*n* = 8) (Wilcoxon*, T =* 36*, z* = −3.51, *p <* 0.001, figure 2*a*). Furthermore, monarch regurgitates harvested after petiole cutting had significantly higher cardenolide amounts than regurgitates collected before petiole cutting on both host plants (*A. curassavica*: Wilcoxon *T* = 55, *z =* −3.748 figure 2*a*, *p <* 0.001; *A. syriaca*: Wilcoxon *T* = 79, *z =* −1.992*, p =* 0.046, figure 2*b*). No differences were found when comparing *Euploea* caterpillar regurgitates obtained before and after vein cutting (Wilcoxon *T =* 63, *z =* 0.752, *p =* 0.463, figure 2*a*).

### (e) Does latex consumption in monarch caterpillars increase butterfly toxicity?

Finally, we compared the cardenolide content of butterflies when reared individually on detached *A. curassavica* leaves (midrib of leaves removed to minimize latex content, *n* = 7; electronic supplementary material, figure S3) with butterflies raised on detached leaves when latex was applied to the caterpillar mouthparts artificially to mimic drinking during the fifth instar (*n* = 6, figure 2*c*). In line with our assumption that latex drinking facilitates sequestration of cardenolides, artificial latex supply increased cardenolide concentrations in adult butterflies (*t*_7_ = −2.66, *p* = 0.033, figure 2*d*; total cardenolide amounts of butterflies differed only marginally; *t*_11_ = −2.08, *p* = 0.062).

### (f) Does latex exposure decrease early-instar survival and is latex consumption limited to late instars?

**Figure 3.**
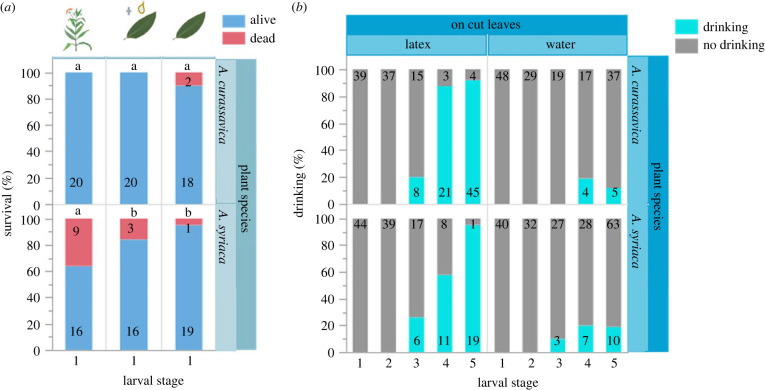
Survival of first-instar caterpillars on milkweed and latex drinking across caterpillar development. (*a*) survival of first-instar *D. plexippus* caterpillars raised on intact plants (left bars), detached leaves with daily application of 1 µl latex (central bars), or detached leaves (right bars) of *A. curassavica* (top) and *A. syriaca* (bottom). Numbers in bars indicate the numbers of caterpillars observed; letters indicate significant differences across treatments. Data on caterpillar survival on intact plants were collected during the experiment shown in figure 1. Please note that only data for the first larval instar are presented here; data for all other larval instars are given in the electronic supplementary material, figure S3. (*b*) Proportions of caterpillars drinking latex of *A. curassavica* or *A. syriaca* (or water as a control) when offered manually across larval development. Caterpillars were raised on detached leaves of the respective milkweed species from which latex was obtained. Initially, *n* = 20 caterpillars per plant and treatment (i.e. 80 caterpillars in total) were observed. Caterpillars were offered latex or water once a day. Numbers in bars indicate the number of observed events per developmental instar.

Classic literature on monarch butterflies suggests that the mortality caused by latex is especially high in neonate caterpillars [10,16,42]. In accordance, when we raised first-instar monarchs on detached *A. syriaca* leaves, we found that mortality was significantly reduced compared to caterpillars grown on intact *A. syriaca* plants (*t*_135_ = −2.52, *p* = 0.039, figure 3*a*; see supplementary material for the other stages, electronic supplementary material, figure S3). However, we found no differences in first-instar caterpillar mortality when reared on intact or detached *A. curassavica* leaves (*t_129_ =* 0.01*, p* = 1, figure 3*a*). Daily application of latex onto first-instar caterpillar mouthparts neither influenced caterpillar survival on detached leaves of *A. curassavica* (*t*_129_ = 0.01, *p* = 1) nor of *A. syriaca* (*t*_135_ = −0.86, *p* = 1).

During the same experiment, we recorded caterpillar responses to latex or water applied to the mouthparts and found a pronounced shift in behaviour between younger and older caterpillars (figure 3*b*; electronic supplementary material, videos S4 and S5). While younger caterpillars (L1–L2) completely rejected latex or water, the frequency of drinking increased over the course of caterpillar development (*F*_4,47.79_ = 6.61, *p* < 0.001). Importantly, caterpillars were more likely to drink latex than water (*F*_1,226.3_ = 136.77, *p* < 0.001). We found no difference in behaviour between milkweed species (*F*_1,221.5_ = 0.02, *p* = 0.898).

## 4. Discussion

In this study, we investigated whether the sabotaging behaviour of monarch caterpillars, traditionally regarded as an adaptation to disarm plant defences, has evolved into a strategy of latex uptake to enhance cardenolide sequestration for protection against predators. The behaviour of monarch caterpillars was compared with that of caterpillars of *Euploea core*, a related milkweed butterfly species that does not store cardenolides for defence. We found several lines of evidence suggesting that late-instar monarch caterpillars do in fact consistently drink latex rather than avoid it. In addition, ingestion of latex through drinking increases the amount of cardenolides sequestered and is therefore likely to result in improved protection against predators [23].

### (a) Behavioural differences suggest divergent strategies between *D. plexippus* and *E. core*

When we compared caterpillar development on the two most important host plants, *A. curassavica* and *A. syriaca*, we found that monarch caterpillars were rather inconsistent in their sabotaging behaviour, especially in older instars. While sabotaging probably increases survival of early instars, particularly on *A. syriaca*, our data strongly suggest that sabotaging is not generally required by monarch caterpillars to avoid impairment by latex. Caterpillars of *Euploea*, on the other hand, showed consistent sabotaging behaviour, supporting the notion that it is a strategy to circumvent the stickiness or toxicity of latex in this non-sequestering species.

Detailed observations of sabotaging behaviour on intact plants revealed striking differences between the last-instar caterpillars of monarch and *Euploea*. While monarch caterpillars eagerly sucked up latex released during petiole cutting, *Euploea* caterpillars thoroughly removed latex exuding from the created furrow. This behavioural difference strongly supports opposing strategies between the two caterpillar species on *A. curassavica* and *A. syriaca*, i.e. *Euploea* caterpillars avoid latex while monarch caterpillars ingest it, most likely to acquire toxins for defence. Active latex uptake by monarch caterpillars and latex avoidance by *Euploea* caterpillars was further demonstrated by the presence of high levels of cardenolides in monarch caterpillar regurgitates after petiole cutting, whereas no increase was detected in *Euploea* regurgitates. Petiole cutting by monarch caterpillars on *A. curassavica* effectively removed latex, as previous work has shown [3]. We note, however, that the latex drinking behaviour may differ on different milkweed species. On *Asclepias humistrata*, a milkweed species with high production of latex and cardenolides, monarch caterpillars have been observed to reduce latex ingestion by removing emerging latex droplets from sabotaged petioles [10]. Since the sequestration of cardenolides in monarchs has been shown to have an upper limit [44], monarch caterpillars may only drink latex until cardenolide saturation is reached and then avoid latex, as has been shown for *A. humistrata*.

Although we have not assessed sabotaging rates, based on our experience over many years of rearing monarch butterflies, we estimate that a last-instar caterpillar will sabotage petioles at least three times per day. Given that the last instar lasts several days, the total amount of cardenolides acquired by latex drinking from *A. curassavica* is likely to be in the order of several hundred micrograms (total amounts from monarch regurgitate samples ranged from 8.46 to 68.77 µg; 32.23 µg mean, ± 6.1 s.e.; note that our samples most likely represented only a fraction of the total foregut contents and actual amounts may have been even higher). Since monarch butterflies reared on *A. curassavica* have been shown to contain average amounts of >600 µg cardenolides (males) or >800 µg cardenolides (females) [45], it is likely that the majority of the sequestered cardenolides are actually derived from latex drinking rather than from leaf tissue consumption. Differences between cardenolide concentrations in regurgitates obtained from monarch caterpillar petiole cuts on *A. syriaca* were less pronounced, most likely due to the much lower cardenolide content of *A. syriaca* compared to *A. curassavica* (including latex) [17]. However, monarch caterpillars may ingest higher amounts of latex on *A. syriaca* plants in the field, which are much larger and probably produce substantially more latex [46] than the pot-grown greenhouse plants used here.

### (b) Coevolutionary implications for the Monarch–Milkweed interaction

The classic literature suggests that sabotaging behaviour protects the monarch caterpillar from latex exudation at the feeding site by deactivating the plant's laticifer system [3,6,42]. Consistent with this notion, reduction of latex flow has been shown to increase survival of first-instar larvae [42]. Accordingly, we found increased mortality of caterpillars on intact *A. syriaca* plants compared to caterpillars reared on detached leaves. However, adding latex did not adversely affect first-instar caterpillars, possibly due to the artificial route of exposure. In *A. curassavica*, by contrast, survival on intact plants, detached leaves and detached leaves with artificial latex supply did not differ. We conclude that sabotaging milkweed by early-instar caterpillars is likely an adaptation to overcome plant defences, but its role in caterpillar survival may differ between milkweed species due to plant species-specific traits such as latex content, latex stickiness and toxicity, or laticifer architecture (branched and non-articulated in *A. syriaca* versus articulated, anastomosing in *A. curassavica*) [28,42]. The latter, in particular, has been reported to be an important predictor of the type of sabotaging behaviour (trenching or vein cutting) in various insect herbivores, with the exception of *Danaus* and *Euploea* caterpillars that show both behaviours on the same plant species [25,26,28].

Our finding that monarch caterpillars change their behaviour from avoiding latex offered with a pipette in young caterpillars to eagerly drinking latex in older caterpillars combines the classical assumption that young caterpillars sabotage to avoid latex with our novel findings showing that older monarch caterpillars actively drink latex during vein cutting. The role of sabotaging and latex drinking behaviour has never been comparatively addressed across the radiation of milkweed butterflies (Danaini). However, the occurrence of sabotaging behaviour in the closely related Ithomiini and many unrelated insects, including dietary generalists [7], suggests that latex avoidance, as found in caterpillars of *Euploea*, represents the ancestral state of the Danaini.

The behavioural differences reported here between monarch and *Euploea* caterpillars are paralleled by divergent physiological adaptations. Whereas monarch caterpillars can tolerate sequestered cardenolides by means of a resistant Na^+^/K^+^-ATPase, caterpillars of *Euploea* naturally consume cardenolide-containing plants but cannot cope with the toxins when injected into their body cavity [47]. Instead, *Euploea* caterpillars degrade cardenolides in the gut lumen, presumably to detoxify them [48]. In the face of antagonistic insect−plant coevolution, monarchs have evolved a number of traits to exploit plant defences (cardenolides) for their own benefit. Here we discovered a novel adaptation of monarchs to milkweed plants and show that toxin uptake can be enhanced by drinking latex [49]. Our study shows that mechanisms of toxin sequestration for defence are not limited to cellular adaptations, but extend to the behavioural level.

## Data Availability

All data underlying our study, as well as statistical code, have been deposited in the Dryad Digital Repository (https://doi.org/10.5061/dryad.qnk98sfns) [50]. There are no restrictions on data availability. Supplementary material is available online [51].
